# LysGR1, a novel thermostable endolysin from *Geobacillus stearothermophilus* bacteriophage GR1

**DOI:** 10.3389/fmicb.2023.1178748

**Published:** 2023-05-19

**Authors:** Dahee Choi, Minsuk Kong

**Affiliations:** Department of Food Science and Technology, Seoul National University of Science and Technology, Seoul, Republic of Korea

**Keywords:** *Geobacillus stearothermophilus*, food spoilage, bacteriophage, endolysin, enzymatically active domain

## Abstract

*Geobacillus stearothermophilus* is a highly thermophilic, spore-forming Gram-positive bacterium that causes flat sour spoilage in low-acid canned foods. To address this problem, we isolated *G. stearothermophilus*-infecting phage GR1 from the soil and characterized its endolysin LysGR1. Phage GR1 belongs to the *Siphoviridae* family and possesses a genome of 79,387 DNA bps with 108 putative open reading frames. GR1 demonstrated a very low degree of homology to previously reported phages, indicating that it is novel. The endolysin of GR1 (LysGR1) contains an N-terminal amidase domain as an enzymatically active domain (EAD) and two C-terminal LysM domains as a cell wall binding domain (CBD). Although GR1 is specific to certain strains of *G. stearothermophilus*, LysGR1 showed a much broader lytic range, killing all the tested strains of *G. stearothermophilus* and several foodborne pathogens, such as *Clostridium perfringens*, *Listeria monocytogenes*, and *Escherichia coli* O157:H7. LysGR1_EAD, alone, also exhibits lytic activity against a wide range of bacteria, including *Bacillus cereus*, which is not terminated by a full-length endolysin. Both LysGR1 and its EAD effectively remove the *G. stearothermophilus* biofilms and are highly thermostable, retaining about 70% of their lytic activity after a 15-min incubation at 70°C. Considering the high thermal stability, broad lytic activity, and biofilm reduction efficacy of LysGR1 and its EAD, we hypothesize that these enzymes could act as promising biocontrol agents against *G. stearothermophilus* and as foodborne pathogens.

## 1. Introduction

*Geobacillus stearothermophilus* is a thermophilic, aerobic, spore-forming bacterium widely distributed throughout environments. A high initial load of *G. stearothermophilus* can cause flat sour spoilage in low-acid canned foods when its spores survive canning and ultra-high temperature processes, followed by germination and outgrowth (Wells-Bennik et al., 2019). In addition, it often forms biofilms in dairy manufacturing plants, which causes significant financial losses in the food industry (Andre et al., 2013; Batt and Tortorello, 2014). Although heat sterilization has been used to control *G. stearothermophilus*, its use is challenging because it can damage the textures, tastes, colors, and nutritional value of foods (Block, 2001). Thus, the development of novel antimicrobial agents against *G. stearothermophilus* has become of interest.

Bacteriophages and phage-derived proteins have gained growing attention as natural antimicrobial agents (Mahony et al., 2020). Phages are viruses found ubiquitously in nature that infect and replicate only in bacterial cells (Stone et al., 2019). At the end of the bacteriophage’s lytic cycle, phages produce peptidoglycan hydrolases, called endolysins, which cleave the cell wall in the host cell to release progeny virions (Fischetti, 2010). Endolysins from Gram-positive bacteria infecting phages generally harbor two distinct domains: a cell wall binding domain (CBD) and an enzymatically active domain (EAD) (Rahman et al., 2021). While EADs cleave the specific bonds constituting the bacterial cell wall, CBDs recognize and bind to the specific receptor of the bacterial cell wall (Loessner et al., 2002). Due to their potent lytic action and proteinaceous nature, endolysins have the potential to be used as food preservatives. Indeed, a number of studies have tested the use of phage endolysins to control diverse food-borne pathogens, including *Staphylococcus aureus*, *Listeria monocytogenes*, *Clostridium perfringens* (Chang et al., 2017; Van Tassell et al., 2017; Cho et al., 2021) in food matrices.

Despite of the importance of *G. stearothermophilus* in food industry, only a few *G. stearothermophilus* phages and their endolysins have been previously reported (Egbert and Mitchell, 1967; Rabussay et al., 1970; Skowron et al., 2018). Jin et al. (2013) isolated a bacteriophage GVE2 infecting *Geobacillus* sp. E263 from a deep-see hydrothermal vent and characterized the roles of its endolysin. Zebrowska et al. (2022) characterized the biochemical properties of a thermostable endolysin of phage TP-84, which infects *G. stearothermophilus*, and tested its potential as a disinfectant. To develop efficient endolysin-based antimicrobials, new *G. stearothermophilus* phages should be isolated and more in-depth studies of their endolysins are required. In this study, we isolated a novel *G. stearothermophilus* phage GR1 and characterized its endolysin LysGR1. Here, LysGR1 and its EAD exhibited much broader lytic activities compared to the high host specificity of GR1. Both recombinant proteins have high thermal stability in the temperature range of −20°C–70°C and effectively removed the *G. stearothermophilus* biofilms. Our findings may facilitate the development of promising biocontrol agents against *G. stearothermophilus*.

## 2. Materials and methods

### 2.1. Bacterial strains and plasmids

The bacterial strains used in this study are listed in Table 1. *Geobacillus stearothermophilus* ATCC 10149 was used for the isolation and propagation of GR1. All *G. stearothermophilus* strains were grown under agitation in Tryptic Soy Broth (TSB) at 50°C. *Bacillus cereus*, *Salmonella*, *Escherichia coli*, and *Shigella flexneri* strains were grown under agitation in Luria–Bertani (LB) broth at 37°C. *Listeria* and *C. perfringens* strains were grown in Brain Heart Infusion (BHI) broth at 37°C with shaking and anaerobic conditions, respectively. *Bacillus* strains, except *B. cereus*, *Cronobacter sakazakii*, *Yersinia enterocolitica*, *Enterococcus faecalis*, *S. aureus*, and *Pseudomonas* strains were grown in TSB at 37°C under agitation. The agar media were generated by supplementation of 1.5% agar with the broth medium. All media were purchased from Difco (Detroit, MI, USA). Bacterial stocks were stored at −80°C with 15% glycerol.

**Table 1:** Antimicrobial activity of phage GR1 and its endolysin LysGR1 and LysGR1_EAD.

	Species	Strain no.^a^	GR1	LysGR1^b^	LysGR1_EAD^b^
Gram-positive	*Geobacillus stearothermophilus*	KCTC 1752	−	**++**	**++**
		KCTC 2107	**+**	**+++**	**+++**
		KCTC 3775	−	**++**	**++**
		ATCC 10149	**+**	**+++**	**+++**
		ATCC 12976	**+**	**++**	**+++**
		ATCC 12978	−	**+++**	**++**
		ATCC 21365	−	**+**	**++**
	*Clostridium perfringens*	NCCP 15911	−	−	−
		ATCC 3624	−	**+**	**+**
		ATCC 13124	−	**++**	**+**
		FD1	−	**++**	**+**
		H3	−	**+**	**++**
		H9	−	**+**	**+**
	*Staphylococcus aureus*	Newman	−	**−**	**−**
	*Listeria monocytogenes*	ATCC 15313	−	**+**	**++**
	*Listeria innocua*	ATCC 33090	−	**+**	**+**
	*Bacillus cereus*	ATCC 10987	−	−	**+**
		NCCP 10841	−	−	**++**
	*Weizmannia coagulans*	KACC 11248	−	**+**	−
	*Bacillus amyloliquefaciens*	KACC 15877	−	**++**	**+++**
	*Bacillus subtilis*	ATCC 23857	−	**+**	**++**
Gram-negative	*Escherichia coli* O157:H7	ATCC 35150	−	**+**	**+**
		ATCC 700728	−	**+**	**++**
	*Cronobacter sakazakii*	ATCC 29544	−	**+**	**+**
	*Salmonella Typhimurium*	ATCC 43147	−	−	N.D.

^a^ATCC, American Type Culture Collection; KCTC, Korean Collection for Type Culture; KACC, Korean Agricultural Culture Collection; NCCP, National Culture Collection for Pathogens; JCM, Japan Collection of Microorganism.

^b^The relative lytic activity was calculated as follows: [OD_600_ control − OD_600_ test] / Initial OD_600_ × 100 (%). -: 0%–10%; +: 11%–40%; ++ 41%–70%; +++: 71%–100%

### 2.2. Isolation of bacteriophage GR1

To isolate *Geobacillus*-infecting phage, a soil sample was collected from the Gyeongchun Line railroad in Seoul, South Korea. A total of 25 g of the solid sample was mixed with 225 mL of Butterfield’s phosphate-buffered dilution water (0.25 M KH_2_PO_4_ adjusted to pH 7.2 with NaOH) in a sterile bag and homogenized using a Bag Mixer 400 CC (Interscience Laboratory Inc., St. Nom, France). Next, 10 mL of the diluted sample was mixed with 10 mL of 2× TSB with 5 mM of MgCl_2_ and CaCl_2_; then, the mixture was incubated after inoculation with 200 μL of the overnight cultured *G. stearothermophilus* ATCC 10149 and shaken for 24 h at 50°C. After incubation, 200 μL of chloroform was added to the overnight cultured mixture, and the mixture was incubated under agitation at 50°C for 10 min. After centrifugation (15,000 × g for 10 min), the supernatant was filtered using a 0.22 μm pore size filter (Sartorius Stedim Biotech, Göttingen, Germany). Then, 10-fold serial dilutions of the filtrate were spotted on *G. stearothermophilus* ATCC 10149 embedded in 0.4% soft TSB agar. The plate was incubated at 50°C for 24 h. To isolate and purify the bacteriophages, the overlay assay was performed as previously described (Kong and Ryu, 2015). The phage resuspension was overlaid on 0.4% soft TSB agar embedded with the host bacterium. This process was repeated at least three times to purify a single phage.

### 2.3. Phage propagation and purification

To propagate the isolated phage, the lysate of a single bacteriophage GR1 plaque was inoculated with the *G. stearothermophilus* ATCC 10149 culture (OD_600_ = 0.5) and incubated at 50°C for 4 h. After centrifugation (15,000 × g at 4°C for 5 min), the supernatant was filtered using a 0.22 μm pore size filter. The filtrate phage particles were concentrated as previously described (Kong and Ryu, 2015), with some modifications. Purified phages were dialyzed in 1 L of SM buffer in snakeskin dialysis tubing (molecular weight cut off (MWCO) 3.5 kDa, Thermo Fisher Scientific, Rockford, USA). The titers of the concentrated phage stocks were determined by the spotting assay and the phage stocks were stored at 4°C for use in further experiments.

### 2.4. Host range analysis

Host range analysis was performed as previously described (Na et al., 2016).

### 2.5. Morphological analysis by transmission electron microscopy

Morphological analysis was performed similarly to a previous study (Lee et al., 2019). Drops of purified phage GR1 stock (about 1.3 × 10^10^ PFU/mL) were placed on the prepared grids and incubated at room temperature for 1 min. After incubation, the excess phage suspension was removed using filter paper and the phage particles were negatively stained with the same volume of 2% uranyl acetate (pH 4.0) for 20 s; the excess solution was removed as described above. GR1 was examined by transmission electron microscopy (Libra 120 model, Carl Zeiss, Oberkochen, Germany) at 120 kV. GR1 was classified according to the International Committee on Taxonomy of Viruses (ICTV) classification.

### 2.6. Genome sequencing and *in silico* analysis

Phage DNA extraction was performed using a Phage DNA Isolation Kit (Norgen Biotek Corporation, Thorold, Ontario, Canada), according to the manufacturer’s instructions. The purified genomic DNA was sequenced using the Illumina Novaseq 6,000 platform at LabGenomics, South Korea, and assembled with a Unicycler assembler v0.4.8 at Sanigen, South Korea. The functions of the predicted open reading frames (ORFs) were determined using BLASTP and InterProScan databases (Altschul et al., 1990). The presence of tRNA-encoding genes was examined using the tRNAscan-SE database. A circular genome map was generated using the CGview server database (Stothard and Wishart, 2005). The amino acid sequence alignments of endolysins were conducted using ClustalX2 (Larkin et al., 2007).

### 2.7. Cloning, expression, and purification of LysGR1 and its EAD, EGFP-CBD fusion protein

The endolysin gene (LysGR1) and its enzymatically active domain (LysGR1_EAD), cell wall binding domain (LysGR1_CBD) were amplified from the genomic DNA of the GR1 phage by the polymerase chain reaction (PCR) (primers used in this study are indicated in Supplementary Table 1). The gene fragments encoding LysGR1 and LysGR1_EAD were treated with NcoI and SalI and cloned into pET28a (Novagen, Madison, WI, USA). The gene fragments encoding LysGR1_CBD were digested with BamHI and SalI and subcloned into pET28a::EGFP (Kong et al., 2015). To purify each protein, a hexahistidine (6 × -His) tag was added to the C-terminus of LysGR1 and its EAD, the N-terminus of the EGFP-CBD fusion protein, respectively. The plasmid with the correct insert was transformed into *E. coli* BL21 (DE3). The expression of the proteins was induced by 0.5 mM of isopropyl β-D-1- thiogalactopyranoside (IPTG) (Sigma-Aldrich, St. Louis, MO), and adjusted the OD_600_ from 0.6 to 0.8, followed by incubation for 20 h at 18°C with agitation. After centrifugation (4,000 × g at 4°C for 15 min), bacterial cells were resuspended in 5 mL of lysis buffer (50 mM Tris-Cl, pH 8.0, 200 mM NaCl). Cells were lysed by sonication (Sonics & Materials, Inc., CT, USA) for 6 min using intervals of 4 s on and 6 s off. To eliminate the insoluble materials, centrifugation was conducted at 21,000 × g and 4°C for 1 h, and the supernatant was collected and purified using 500 μL of nickel–nitrilotriacetic acid (Ni–NTA) agarose (Qiagen, Hilden, Germany). The concentration of proteins was examined by Bradford assay, while the mass, purity, and solubility were assessed by sodium dodecyl sulfate-polyacrylamide gel electrophoresis (SDS-PAGE). Purified LysGR1 and LysGR1_EAD were stored in lysis buffer at −80°C, while purified EGFP-CBD was stored in storage buffer (50 mM Tris-Cl (pH 8.0), 200 mM NaCl, 50% glycerol) at −20°C, respectively.

### 2.8. Turbidity reduction assay

Bacterial cells were grown to the exponential phase (approximately OD_600_ = 0.6) and resuspended in reaction buffer (20 mM Tris-Cl, pH 8.0). The purified endolysin (LysGR1) and its EAD were added to a final concentration of 1.6 μM, and the OD_600_ values were monitored over time at 45°C using a SpectraMax i3x plate reader (Molecular Devices, Sunnyvale, CA). The relative lytic activity was calculated when the OD_600_ of the endolysin-treated group (experimental OD_600_) reached the lowest value using the equation as follows:

$$\frac{Control\;OD_{600}-Experimental\;OD_{600}}{Initial\;OD_{600}}\times 100\;\left(\%\right)$$

The effect of pH on the enzymatic activity of LysGR1 and LysGR1_EAD was assessed, as previously described (Ha et al., 2018), yet with some modifications. An amount of 1.6 μM of endolysin was added to *Bacillus amyloliquefaciens* KACC 15877 cells suspended in Britton–Robinson universal buffer (0.04 M H_3_PO_4_, 0.04 M H_3_BO_3_, 0.04 M CH_3_COOH, and 0.2 M NaCl) with various pH buffers, which ranged between 6 and 10. To evaluate the effect of the temperature on LysGR1 and its EAD lytic activity, preincubated LysGR1 and its EAD at different temperatures (−20–80°C for 15 min) were used. The effect of NaCl on the enzymatic activity of LysGR1 and its EAD was tested with the reaction buffer using various NaCl concentrations (0–1,000 mM).

### 2.9. Time-killing assay

The time-killing assay was conducted as previously described with some modifications (Lee et al., 2021). The exponentially growing *G. stearothermophilus* ATCC 10149 cells (OD_600_ = 0.6) were washed once and resuspended to OD_600_ = 1.0 by PBS buffer and treated with 1.6 μM of LysGR1 and EAD, respectively. PBS buffer used as a control. After treatment, the mixture was incubated at 50°C for 1 h. The cell aliquots were harvested at the indicated time points (0, 10, 60 min) and diluted, plated in tryptic soy agar plates for enumeration. The number of viable cells was assessed by counting the colonies, and time-killing assay was performed in triplicate.

### 2.10. Biofilm reduction assay on 24-well polystyrene surface

The biofilm removal assay was performed as previously described, although with some modifications (Cha et al., 2019). To determine the biofilm reduction capacity of LysGR1 and LysGR1_EAD, the *G. stearothermophilus* ATCC 12980 (= KCTC 1752) strain was incubated in TSB medium and sub-cultured into each well of a 24-well polystyrene microplate to generate biofilms. After incubation of the microplate for 48 h at 50°C, each well was washed at least three times with PBS, and the washed plate was incubated for 30 min at 50°C to fix the biofilms. After fixing the biofilms, serially diluted LysGR1 and LysGR1_EAD were added to each well, in PBS, to a final volume of 1 mL per well, while PBS was also used as the negative control. The microplate was incubated for 1 h at 50°C, and each well of the microplate was washed three times with PBS, then, stained with 0.1% crystal violet solution. The excess crystal violet solution was removed through additional washing with PBS, and the stained biofilms were solubilized with 33% acetic acid. The absorbance of bacteria in the biofilm was measured at 570 nm, and the remained biomass was presented as the A570 value. The relative A570 was calculated as follows: the A570 test (endolysin added or buffer only) divided by the A570 control (buffer only).

### 2.11. EGFP-fusion LysGR1_CBD binding assay

The binding capacity of the EGFP fusion LysGR1_CBD (EGFP-CBD) was assessed, as previously described (Loessner et al., 2002). Both an LSM 800 (Carl Zeiss, Germany) and an Eclipse Ti2-E fluorescent microscope (Nikon, Japan) were used in this assay.

### 2.12. Accession numbers

The GenBank accession numbers of GR1 and LysGR1 are OK896991 and UDY80750, respectively.

## 3. Results

### 3.1. Isolation and characterization of phage GR1

**Figure 1:**
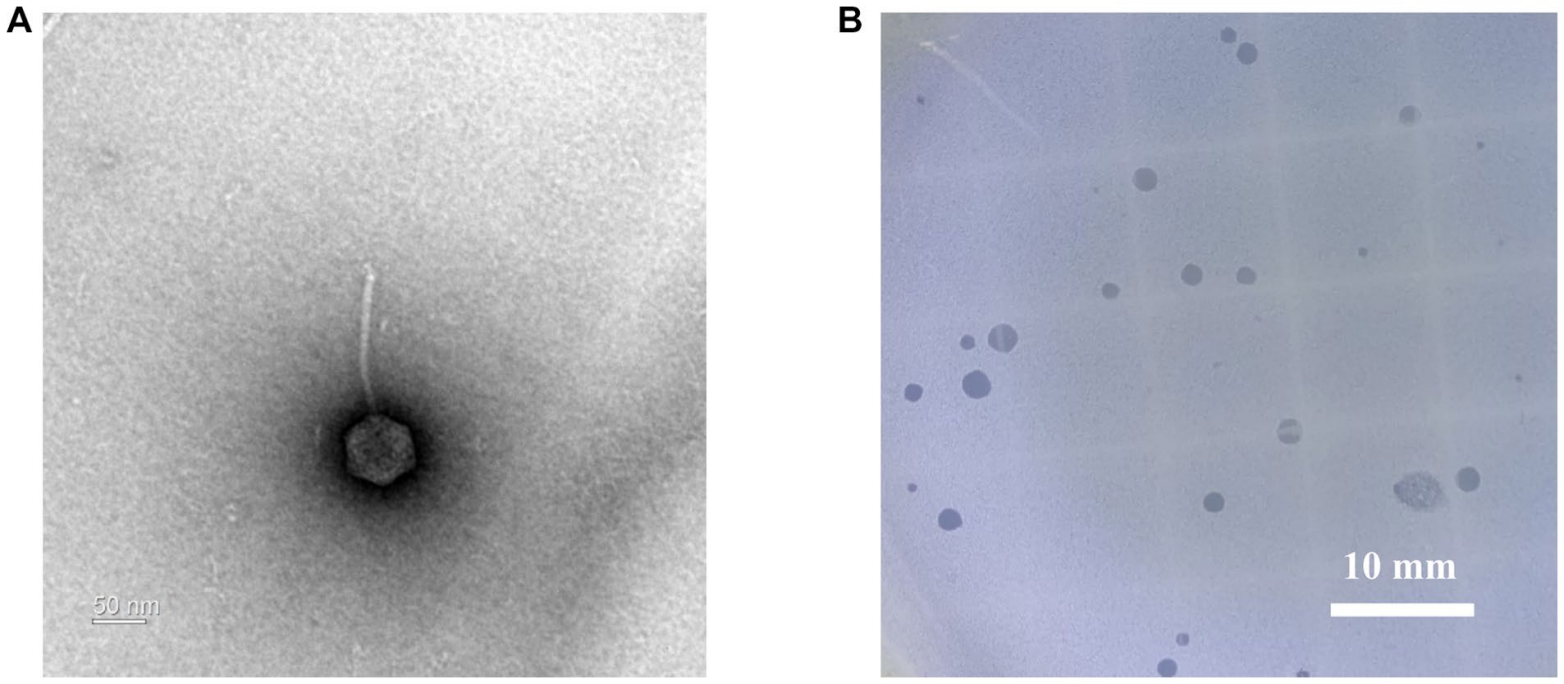
Morphological analysis of *G. stearothermophilus* infecting phage GR1. **(A)** A transmission electron microscopy image of GR1. **(B)** Plaque morphology of GR1.

*Geobacillus stearothermophilus* phage GR1 was isolated from roadside soil, in Seoul, using *G. stearothermophilus* ATCC 10149 as a host. Transmission electron microscopy (TEM) showed that GR1 belongs to the *Siphoviridae* family, which has an icosahedral head with a diameter of 88 ± 9 nm (n = 10) and a non-contractile tail with a length of 187 ± 44 nm (n = 10) (Figure 1A). GR1 produced large, clear plaques against its host strain (Figure 1B) and infected three out of the seven strains of *G. stearothermophilus* (Table 1). GR1 did not infect other Gram-positive or Gram-negative bacteria, indicating that it has a high host specificity.

**Figure 2:**
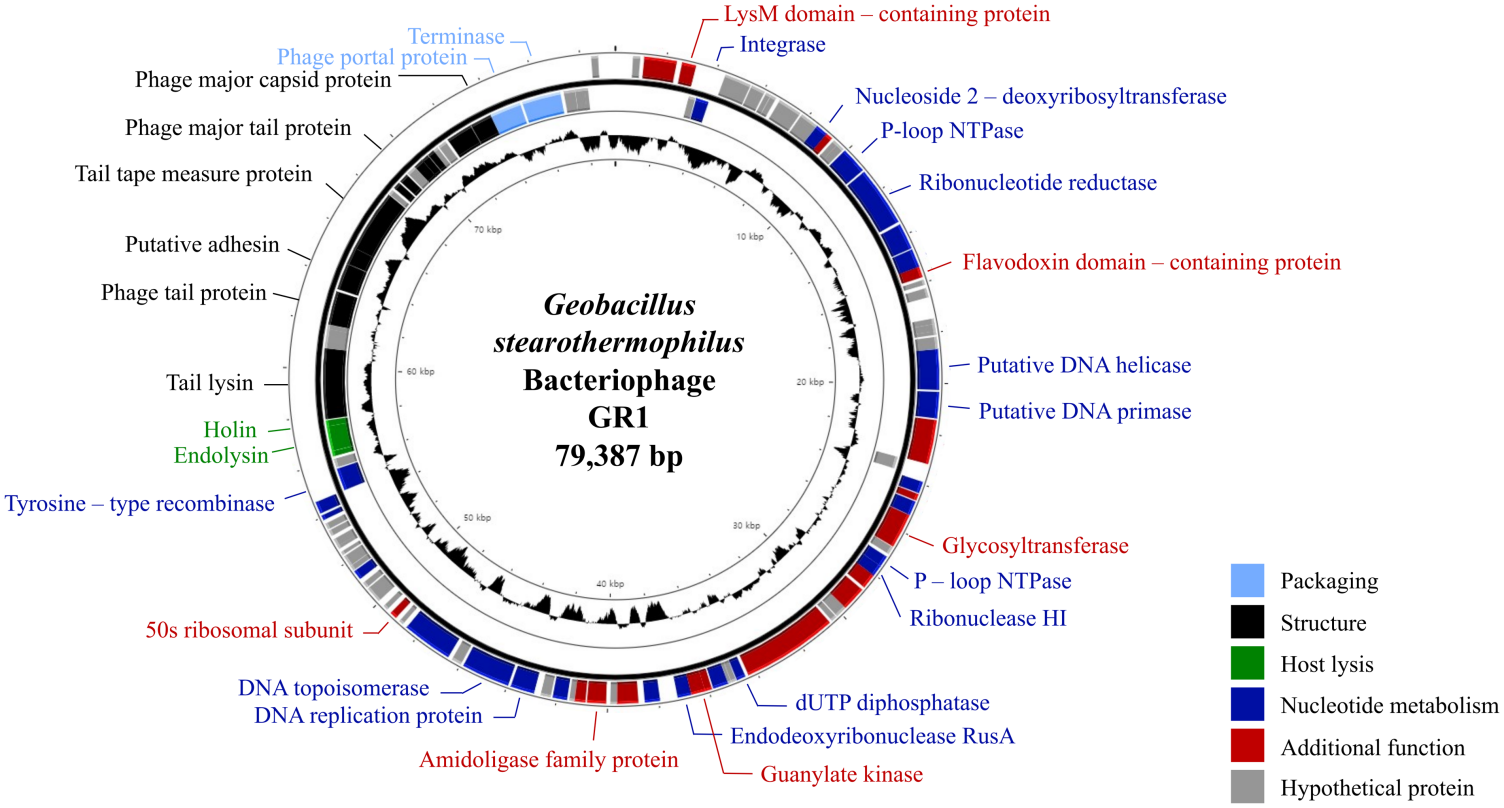
The genome map of GR1. Each ORF color indicates its function: deep blue: nucleotide metabolism; sky blue: packaging; green: host lysis; black: structural proteins; red: additional functions; grey: hypothetical proteins. The inner circle with the black graph shows the GC contents.

The complete genome of phage GR1 comprises 79,387 bps of double-stranded DNA, with a 32.34% average G + C content, 108 putative open reading frames (ORFs), and 1 tRNA. The functional ORFs were categorized into six different groups according to their function: phage DNA packaging, phage structure, host lysis, nucleotide metabolism, additional function, and hypothetical proteins (Figure 2). The presence of an integrase and a tyrosine-type recombinase suggest that GR1 is a temperate phage. Other genes encoding the major capsid protein, putative adhesin, and terminase large subunit were also identified in the phage. BLASTN analysis revealed that there are no genetically similar phages, indicating that GR1 is a novel phage.

### 3.2. Identification of endolysin LysGR1 and its EAD

**Figure 3:**
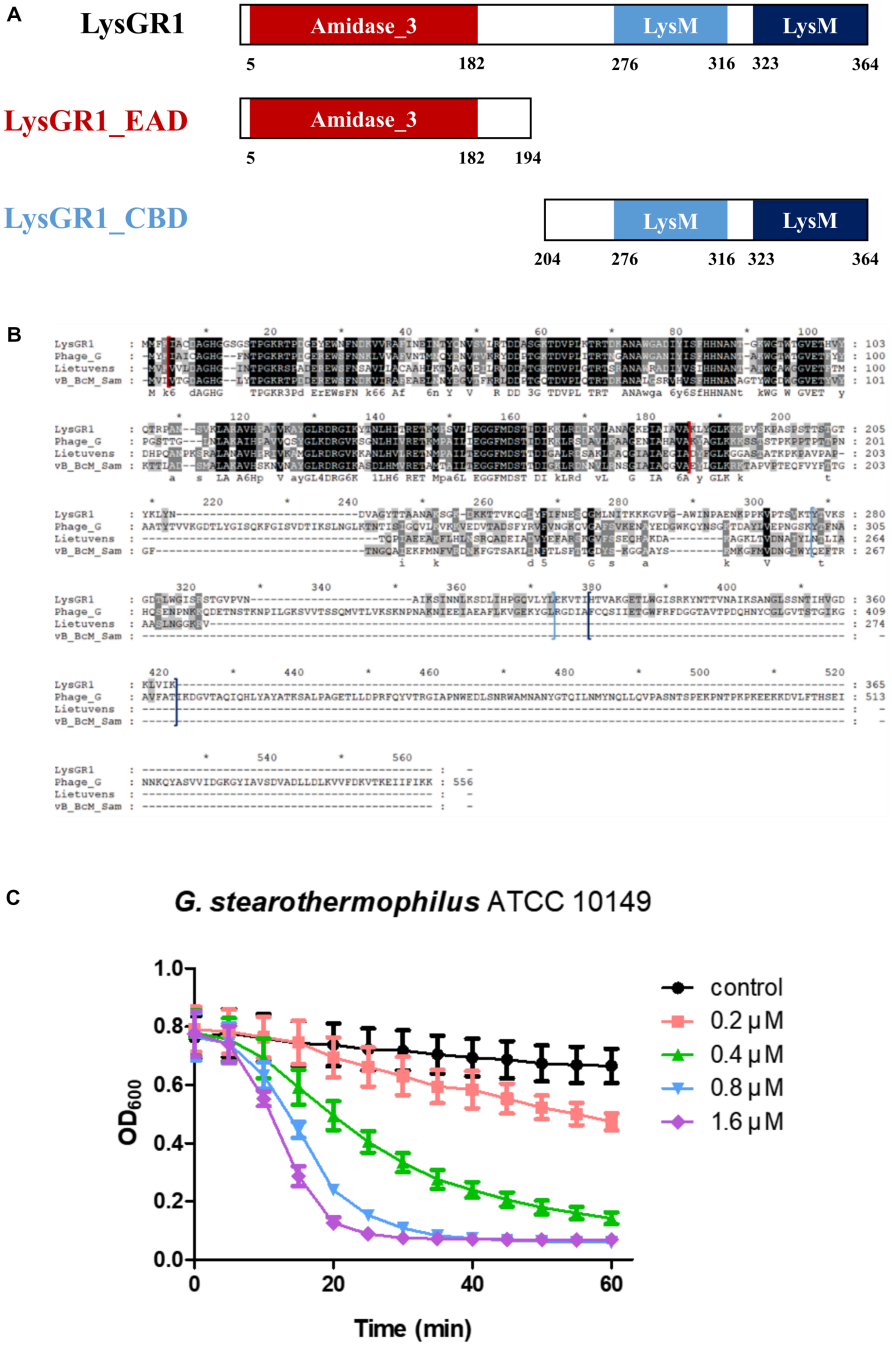
Modular structure of a novel endolysin LysGR1. **(A)** Schematic representation of LysGR1 and its EAD and CBD. LysGR1 has a conserved amidase domain as an EAD at the N-terminus and tandem repeats of LysM motifs as a putative CBD at the C-terminus. LysGR1_EAD contains only an amidase-3 domain, and the CBD construct contains two LysM domains. **(B)** Amino acid sequence alignment of LysGR1 with other endolysins from *Bacillus* and *Sporosarcina* infecting phages. Phage_G: N-acetylmuramoyl-L-alanine amidase from *Bacillus megaterium* phage G, Lietuvens: N-acetylmuramoyl-L-alanine amidase from *Sporosarcina* phage Lietuvens, vB_BcM_Sam: N-acetylmuramoyl-L-alanine amidase from *Bacillus* phage vB_BcM_Sam112. Each domain of LysGR1 indicated with colored square bracket: red: Amidase_3; light blue: first LysM; dark blue: second LysM. Conserved residues are shaded in black and gray (dark gray: > 70% conserved; light grey: > 40% conserved). **(C)** Dose-dependent lytic activity of recombinant LysGR1 against *G. stearothermophilus* ATCC 10149 cells.

The putative endolysin gene was identified in the genome of the bacteriophage GR1 and named LysGR1. Pfam and BLASTP analyses revealed that LysGR1 is a putative N-acetylmuramoyl-L-alanine amidase and contains an N-terminal amidase_3 domain (PF01520) as an enzymatically active domain (EAD), and C-terminal tandem repeats of LysM motifs (PF01476) as a putative cell wall binding domain (CBD) (Figure 3A). Amino acid sequence analysis revealed that LysGR1 only shares 27%, 32%, and 34% identities with lysins from the *Bacillus megaterium* phage G (GenBank protein ID: YP_009015333.1), *Sporosarcina* phage Lietuvens (GenBank protein ID: QIG62545.1), and *Bacillus* phage vB_BcM_Sam112 (GenBank protein ID: QGF21730.1), respectively (Figure 3B), which confirms its novelty. To test the lytic activity, we cloned the recombinant LysGR1 and its EAD (amino acids 1–194, denoted hereafter as LysGR1_EAD) to contain a C-terminal His-tag. Both proteins were expressed in their soluble form in *E. coli* and were purified by Ni-NTA affinity chromatography, which resulted in a homogeneous preparation (Supplementary Figure 1). The protein yield of LysGR1 was relatively lower than LysGR1_ EAD: 0.6 mg of LysGR1 vs. 5 mg of its EAD from 50 mL of *E. coli* cell lysates. A turbidity reduction assay showed that the lytic activity of LysGR1 against *G. stearothermophilus* ATCC 10149 cells is dose-dependent (Figure 3C), while it also exhibited a broad lytic spectrum against all the tested *G. stearothermophilus* strains (Table 1). In addition to *G. stearothermophilus,* LysGR1 killed various *Bacillus* spp. including, *C. perfringens*, *L. monocytogenes*, *E. coli* O157:H7, and *C. sakazakii* (Supplementary Figure 2).

### 3.3. Bacteriolytic and bactericidal activity of LysGR1 and EAD

**Figure 4:**
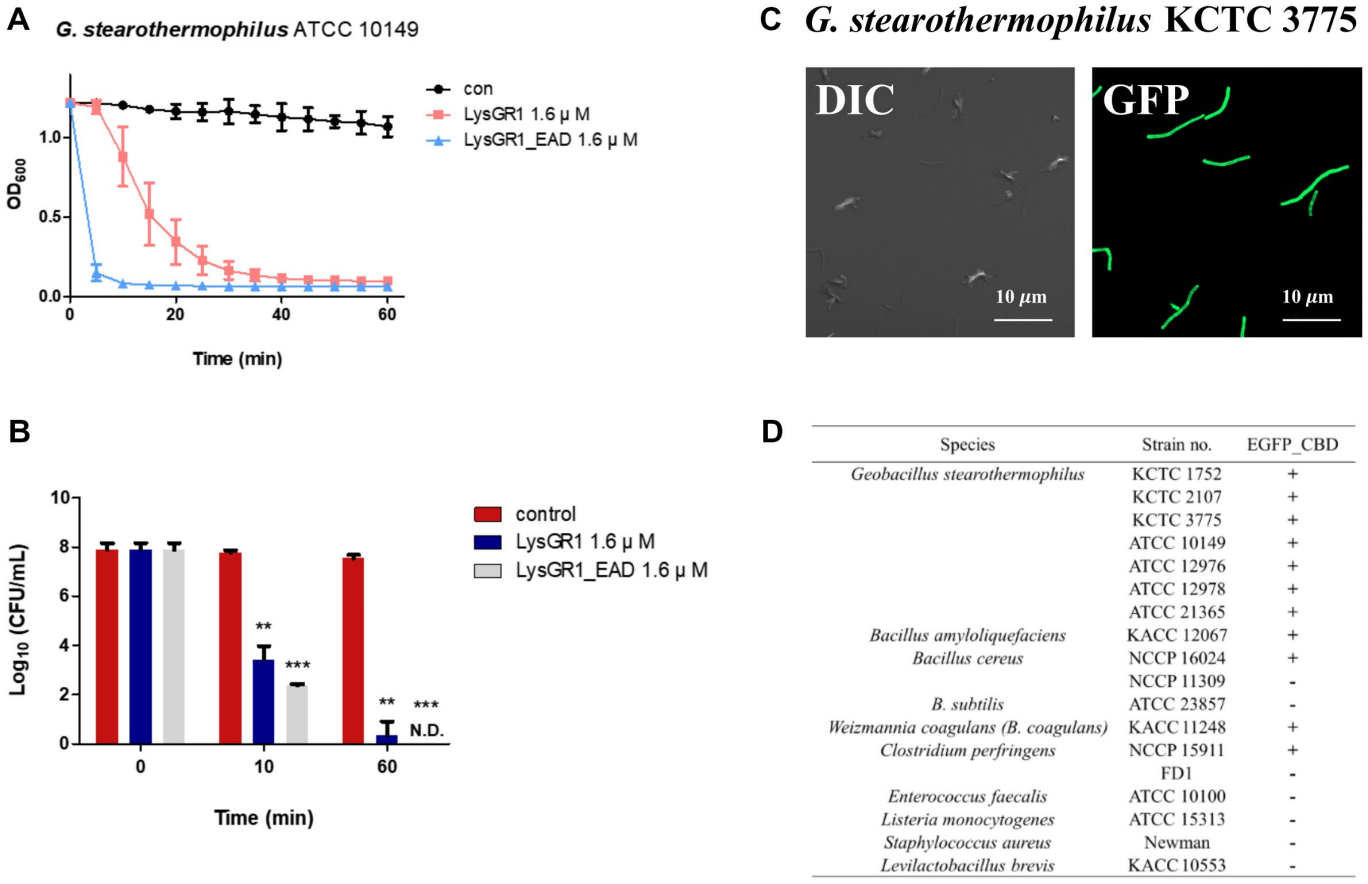
Antimicrobial and binding activity of LysGR1 and its domains. **(A)** Comparisons of the lytic activity of LysGR1 and its EAD against *G. stearothermophilus* ATCC 10149 cells. **(B)** Time-killing activity of LysGR1 and EAD against *G. stearothermophilus* ATCC 10149 cells was determined in PBS buffer. The error bars indicate the standard deviation and data were replicated for three independent times and analyzed based on the PBS results by *t*-test, *p < 0.05, **p < 0.01, ***p < 0.005. **(C)** Binding capacity of LysGR1_CBD toward *G. stearothermophilus* KCTC 3775 cells. **(D)** Binding spectrum of LysGR1_CBD.

To determine the lytic and antimicrobial activity of LysGR1 and its EAD, the turbidity reduction assay and time-killing test were conducted, respectively. Compared to LysGR1, LysGR1_EAD also possesses a similar lytic range to LysGR1, but interestingly, it generally lyses bacterial cells more rapidly than its parental endolysin (Figure 4A). Moreover, LysGR1_EAD can lyse several *B. cereus* strains, which LysGR1 could not kill (Table 1). We also measured the number of viable bacterial cells to evaluate the bactericidal ability of LysGR1 and its EAD in a time-kinetic manner (Figure 4B). LysGR1 and EAD caused 4.4 and 5.4 log reduction, respectively, of *G. stearothermophilus* cells after the addition of 1.6 μM of each enzyme for 10 min at 50°C. At 1 h, no viable cells were detected after the treatment of EAD, while LysGR1-treated group showed 7.2 log reduction, indicating the antimicrobial potential of both LysGR1 and EAD.

### 3.4. Binding activity of LysGR1_CBD

To examine the binding ability of LysGR1, we fused EGFP to the C-terminal tandem repeats of LysM domains of LysGR1 (amino acids 204–364, hereafter called LysGR1_CBD). The fluorescence binding assay revealed that LysGR1_CBD could bind to all *G. stearothermophilus* strains tested (Figures 4C,D), proving the presence of CBD within LysGR1. LysGR1_CBD also binds to some strains of *B. amyloliquefaciens*, *B. cereus*, *W. coagulans*, and *C. perfringens cells*, suggesting that LysGR1_CBD targets broadly conserved cell wall structure of these bacteria.

### 3.5. Biochemical properties of LysGR1 and its EAD

**Figure 5:**
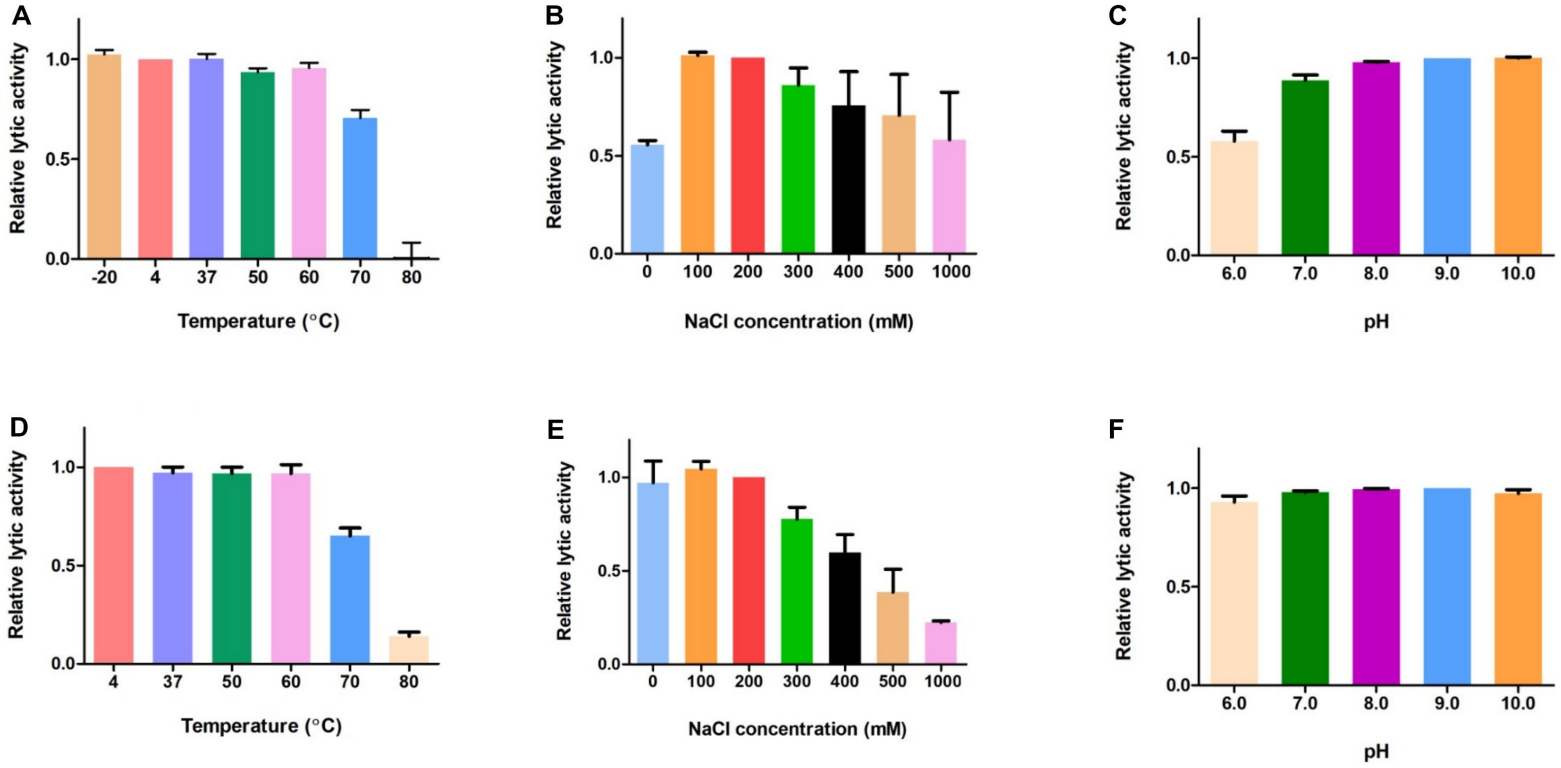
Biochemical properties of LysGR1 and its EAD. The effects of temperature **(A)**, NaCl **(B)**, and pH **(C)** on the lytic activity of LysGR1 and the effects of temperature **(D)**, NaCl **(E)**, and pH **(F)** on the lytic activity of LysGR1_EAD against *B. amyloliquefaciens* KACC 15877 cells. Each column represents the mean of triplicate experiments, and the error bars indicate the standard deviation.

Next, we evaluated the effects of temperature, NaCl, and pH on the lytic activity of LysGR1 and its EAD. The thermal stability was analyzed following the pre-incubation of the enzymes at each temperature for 15 min. Both LysGR1 and its EAD were not significantly affected by temperatures up to 60°C and retain more than 60% lytic activity after incubating at 70°C (Figures 5A,D), indicating their high thermal stabilities. Both enzymes also showed the highest lytic activity at 100–200 mM, while LysGR1_EAD was more sensitive to increasing NaCl concentrations than LysGR1 (Figures 5B,E). Furthermore, LysGR1 and its EAD were relatively stable under a wide range of pHs (pH: 6.0–10.0) (Figures 5C,F).

### 3.6. Biofilm reduction activity of LysGR1 on 24-well polystyrene surface

**Figure 6:**
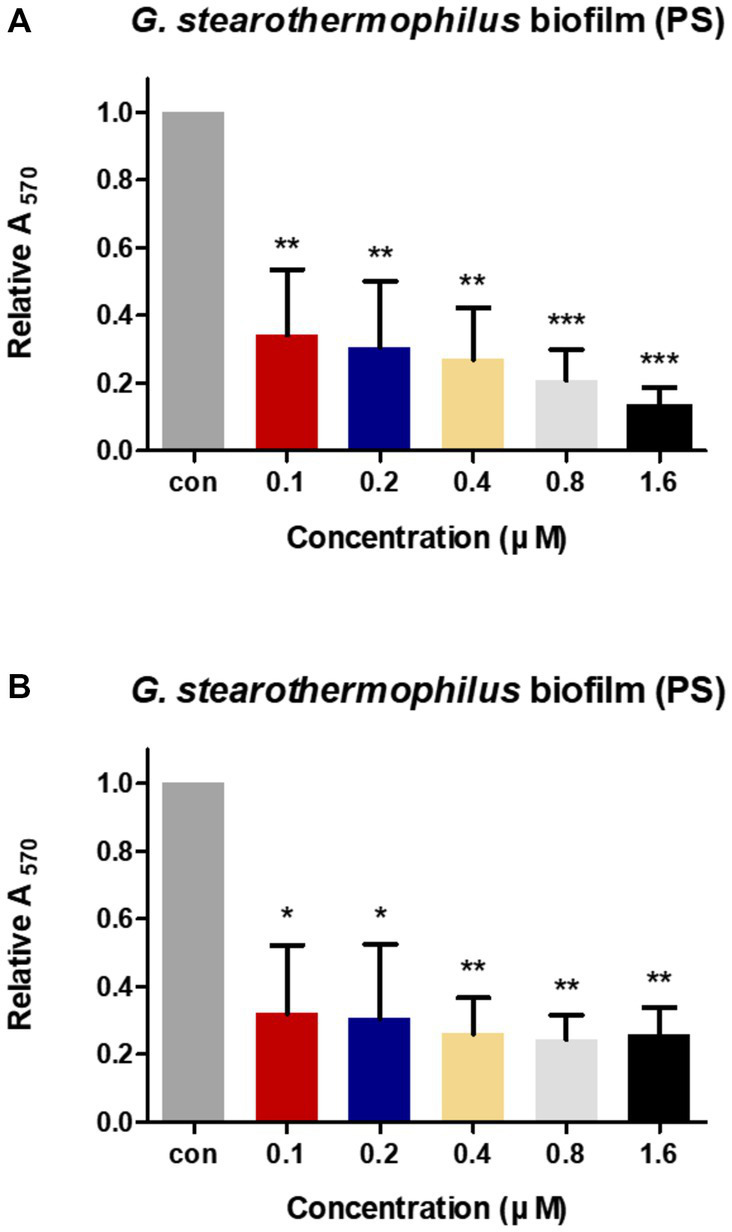
Biofilm reduction activity of LysGR1 **(A)** and its EAD **(B)** in 24-well polystyrene microplates. *G. stearothermophilus* ATCC 12980 biofilms on the surface of polystyrene were treated with various concentrations of LysGR1 and its EAD for 1 h. Each column represents the mean of triplicate experiments, and the error bars indicate the standard deviation. Data were analyzed based on the buffer-treated negative control by *t*-test, *p < 0.05, **p < 0.01, ***p < 0.005.

The anti-biofilm activities of LysGR1 and LysGR1_EAD were tested against the *G. stearothermophilus* ATCC 12980 biofilms, which had been formed in 24-well polystyrene microplates. Both LysGR1 and its EAD showed dose-dependent biofilm reduction efficacies (Figures 6A,B). A total of 86.86 and 74.27% of the biofilms were removed following treatment with 1.6 μM LysGR1 and its EAD, respectively. This result suggests that LysGR1 and LysGR1_EAD could be used as natural anti-biofilm agents against *G. stearothermophilus*.

## 4. Discussion

*Geobacillus stearothermophilus* is a thermophilic, aerobic, spore-forming bacterium with a growth temperature range of 37°C–75°C (Nazina et al., 2001). Usually, *G. stearothermophilus* is responsible for flat-sour spoilage in low-acid canned foods because it produces heat-stable proteases, lipases, and short-chain fatty acids that sour the product without producing gases (Batt and Tortorello, 2014). *Geobacillus stearothermophilus* is also especially problematic in powdered milk processing because general thermal treatments are inefficient in the removal of highly heat-resistant spores (Burgess et al., 2010). During evaporation, the milk is exposed to temperatures between 45°C and 75°C, which are suitable for the germination and growth of residual *G. stearothermophilus* spores in manufacturing plants (Ruckert et al., 2004; Kumar et al., 2021). Furthermore, *G. stearothermophilus* is able to form biofilms resistant to cleaning chemicals and sanitizers, thus, becoming a continuous source of food contamination (Flint et al., 2001). Therefore, there is increasing demand to develop antimicrobial agents against *G. stearothermophilus*.

In this study, we isolated the *Geobacillus*-infecting bacteriophage GR1 and characterized its endolysin LysGR1 and its EAD. GR1 has 79,387 bps of DNA, which represents the second largest genome size among *Geobacillus* phages, after the *Geobacillus* virus E3 (141,298 bp). BlastN analysis revealed that GR1 has a very low sequence similarity to previously reported *Geobacillus* phages indicating its novelty. LysGR1 shares some amino acid sequence similarities with other bacterial autolysins, although the overall identity is around 50% due to the low sequence homology of the CBD region. Interestingly, phage GR1 can only infect a few strains of *G. stearothermophilus*, whereas, LysGR1 and its EAD showed a much broader lytic range, including *B. amyloliquefaciens*, *Bacillus subtilis*, *L. monocytogenes*, *C. perfringens*, and are even capable of killing some Gram-negative bacteria without any pretreatment with outer membrane permeabilizers. In the case of Gram-negative bacteria-targeting endolysins, cationic peptides, such as poly-L-Arg and ε-poly-L-lysine, have been fused to endolysins to destabilize the negatively charged outer membranes of Gram-negative bacteria, which increases the chance of the lysins accessing the peptidoglycan layer (Gutierrez and Briers, 2021). Considering that LysGR1 has a net positive charge (pI = 9.75), owing to the high proportion of lysine residues (41/365 aa), we hypothesize that this basic property might partially work as an outer membrane permeabilizer. LysGR1 and its EAD also showed a similar lytic range and effectively removed the *G. stearothermophilus* biofilms on the polystyrene surface (over 75% of the biofilms). Compared to the EAD, LysGR1 tends to be more effective at lysing *G. stearothermophilus* cells (ATCC 12978, KCTC 3775) that are strongly bound with LysGR1_CBD. On the other hand, bacterial cells that have weak or no binding toward LysGR1_CBD are generally more susceptible to EAD than the full-length endolysin. These CBD-independent, high lytic activity of the EAD might arise from a positive net charge (pI = 9.54) and a small size that efficiently enable the penetration of EAD to peptidoglycan mesh (Demchick and Koch, 1996; Low et al., 2011). In this case, CBD rather hinders the hydrolytic activity of EAD when the CBD has no or less cognate target by enlarging the size of entire enzyme which is undesirable feature. It is also possible that EAD has its own cell wall binding ability as shown in a previous study (Mayer et al., 2011), which undoubtedly needs further experiments.

Although endolysins are considered promising eco-friendly antimicrobials due to their rapid action, specificity, and low risk of bacterial resistance, their low stabilities often pose a problem in their applications to the food industry (Lee et al., 2022). Indeed, endolysins from mesophilic bacteria infecting phages generally lose their hydrolytic function above 45°C–60°C (Heselpoth et al., 2015a; Oliveira et al., 2016; Ni et al., 2021). To address this, one research group tried to fuse the catalytic domains of thermophile lytic enzymes to the cell wall binding domains of *C. perfringens* endolysins to create an anti-clostridium agent (Swift et al., 2015, 2019). Alternative approaches, such as structure-based point mutations (Heselpoth et al., 2015b; Love et al., 2021) the *in silico* design of chimeric endolysins (Haddad Kashani et al., 2017) or stabilizer addition (Heselpoth et al., 2015a) have also been reported to improve the thermostability of the endolysins. Biochemical analysis revealed that LysGR1 and its EAD are highly thermostable, maintain their full lytic activity from 4°C to 60°C, and even retain over 60% of their lytic activity after incubation at 70°C for 15 min. In addition, LysGR1_EAD has a high protein yield (100 mg/L) through the *E. coli* T7-lac expression system, potentiating its possible use as a fusion partner for generating heat-stable chimeric antimicrobials. Overall, by possessing broad lytic activities and high stabilities, both LysGR1 and EAD could be promising biocontrol agents against both food-borne pathogens and food spoilage bacteria.

## Data availability statement

The data presented in the study has been deposited in the GenBank database, under accession number OK896991.

## Author contributions

DC and MK conceived and designed the experiments and wrote the manuscript. DC performed the experiments and analyzed the data. All authors contributed to the article and approved the submitted version.

## Funding

This work was supported by the National Research Foundation of Korea (NRF) grant funded by the Korean government (MSIT) (No., no. 2020R1C1C1008127).

## Conflict of interest

The authors declare that the research was conducted in the absence of any commercial or financial relationships that could be construed as a potential conflict of interest.

## Publisher’s note

All claims expressed in this article are solely those of the authors and do not necessarily represent those of their affiliated organizations, or those of the publisher, the editors and the reviewers. Any product that may be evaluated in this article, or claim that may be made by its manufacturer, is not guaranteed or endorsed by the publisher.
