# Supplementary material for: LysGR1, a novel thermostable endolysin from Geobacillus stearothermophilus bacteriophage GR1
Source: Front Microbiol. 2023 May 19;14:1178748. doi: 10.3389/fmicb.2023.1178748 (PMC10237291; doi:10.3389/fmicb.2023.1178748)

Supplementary Material


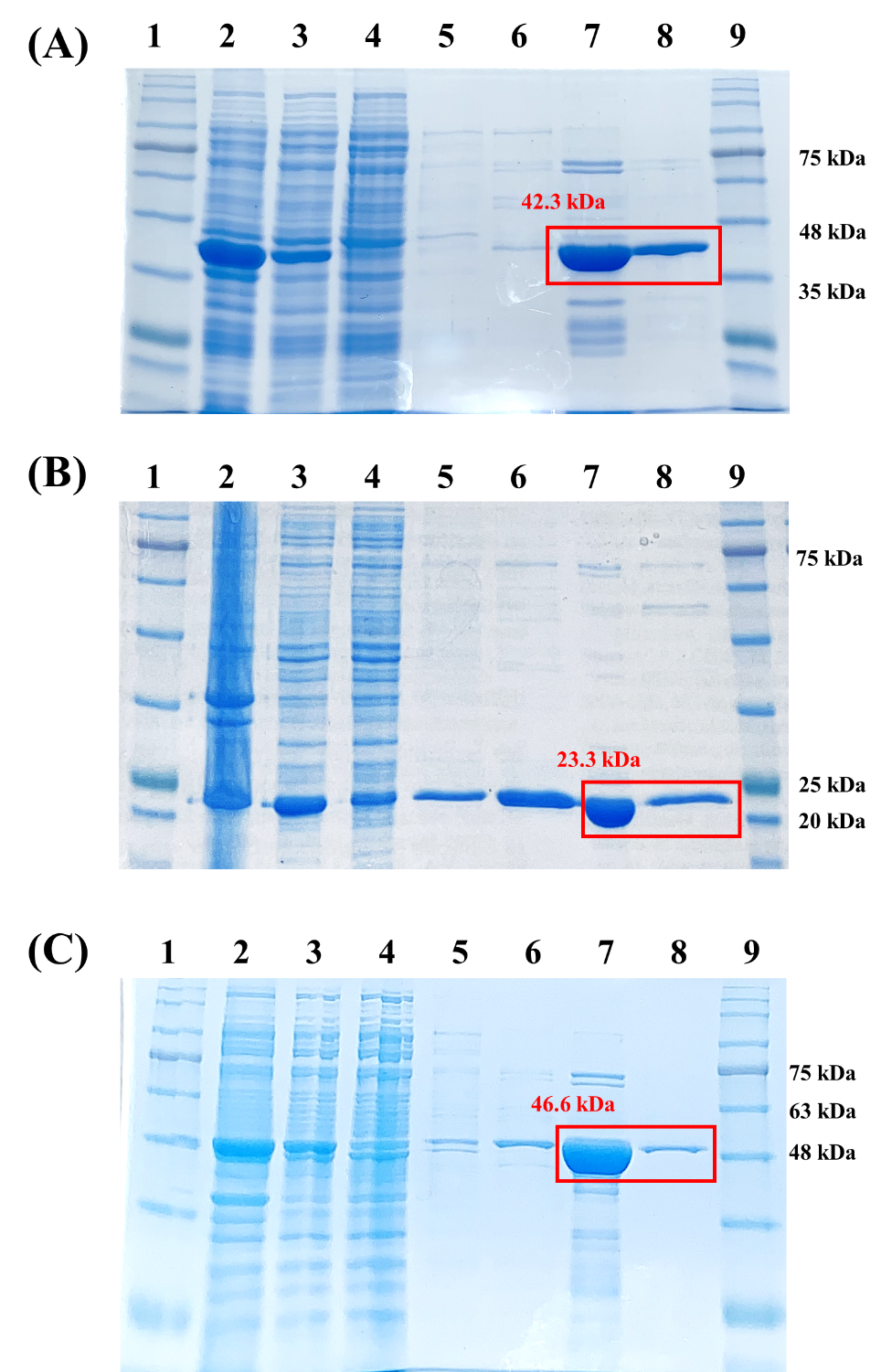


**Supplementary Figure 1.** SDS-PAGE analysis of purified LysGR1 (42.3 kDa) (**A**), its EAD (23.3 kDa) (**B**), and EGFP-fused LysGR1_CBD (46.6 kDa) (**C**). Lane 1 & 9; protein marker (GenDepot, P8503-050), lane 2; cell extract, lane 3; supernatant, lane 4; Ni-NTA flow-through, lane 5; washing (10 mM imidazole), lane 6; washing (20 mM imidazole), lane 7 & 8; elution (200 mM imidazole). Red boxes indicate the target proteins we used.


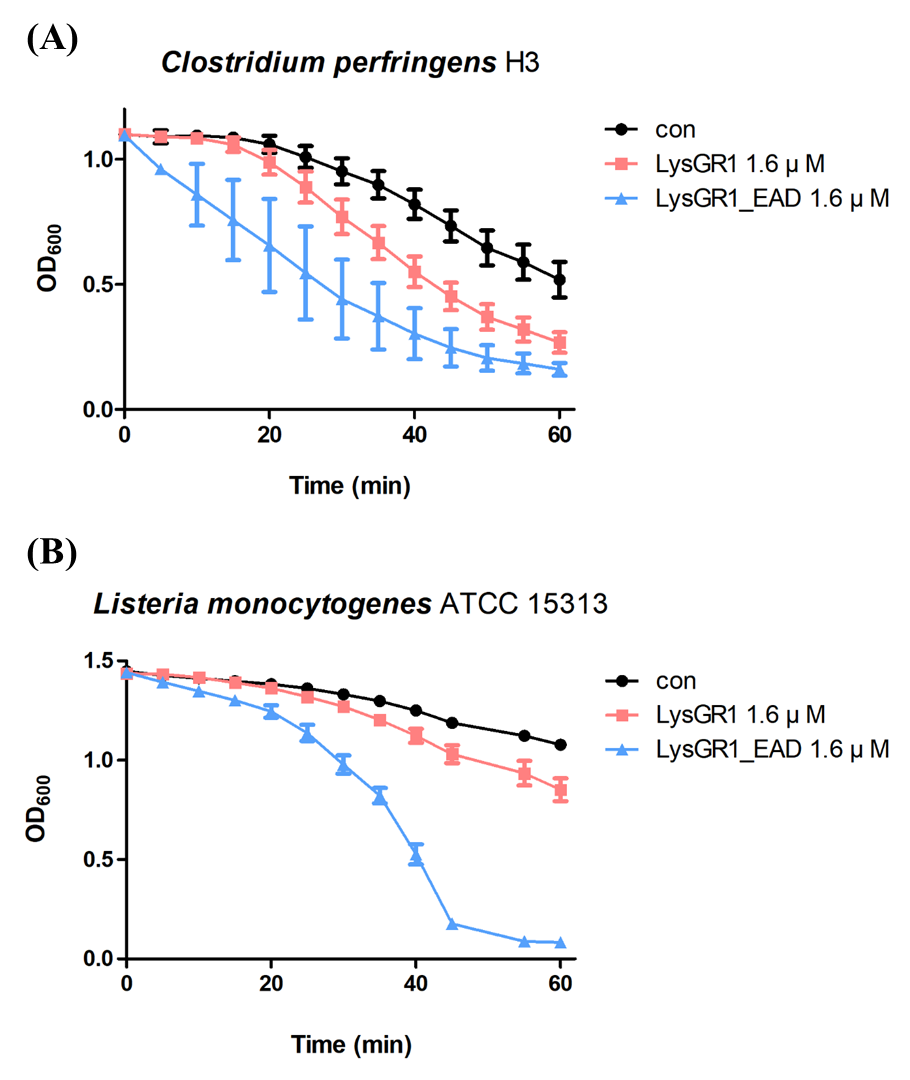


**Supplementary Figure 2.** Lytic activity of LysGR1 and LysGR1_EAD against *Clostridium perfringens* H3 cells (A) and *Listeria monocytogenes* ATCC 15313 cells (B).

**Supplementary Table 1** Plasmids and primers used in this study


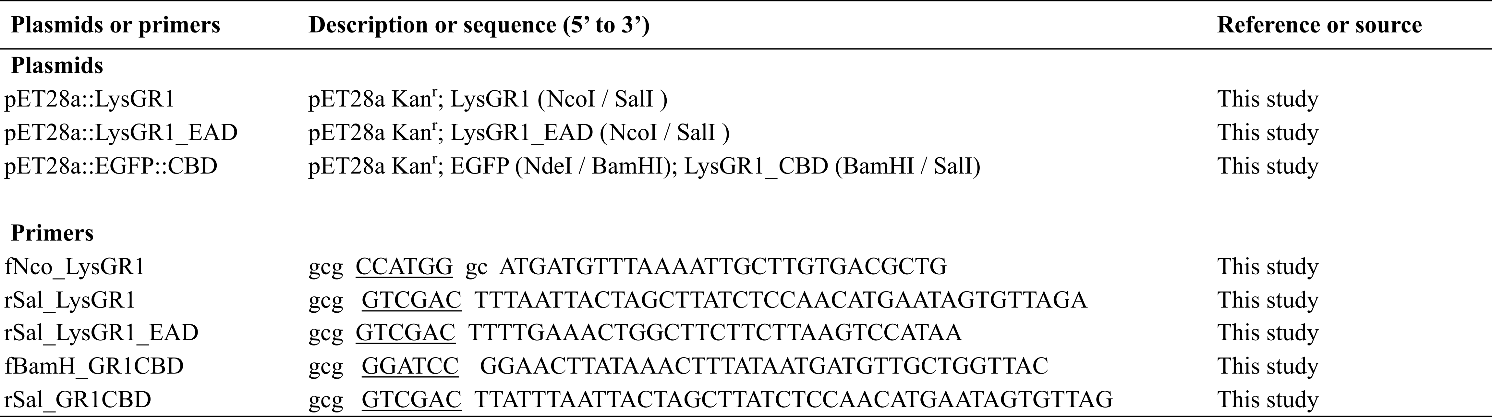

Supplement: Supplementary file 1 [file Data_Sheet_1.docx]
